# YOLO-Net: A lightweight edge-enhanced detection model for small-object recognition in tennis match scenarios

**DOI:** 10.1371/journal.pone.0335558

**Published:** 2026-07-14

**Authors:** Xiangyu Du, Tao Wang, Weiwei Zu, Xianjing Dong, Liangquan Jia

**Affiliations:** 1 School of Physical Education and Health, Guangdong Polytechnic Normal University, Guangzhou, China; 2 School of Information Engineering, Huzhou University, Huzhou, China; 3 School of Tourism and Public Administration, Huzhou Vocational and Technical College, Huzhou, China; Nanjing Forestry University, CHINA

## Abstract

The rapid advancement of deep learning has enabled intelligent analysis in professional sports, yet tennis remains particularly challenging due to small and fast-moving objects, frequent occlusions, and complex backgrounds. To address these difficulties, we propose YOLO-Net, a lightweight detection framework tailored for tennis event analysis. Built upon YOLO11n, the framework integrates three task-oriented improvements: a C3k-MSEIS module for multi-scale edge enhancement and dual-domain feature selection to refine fine-grained boundaries; an ECA channel attention mechanism inserted after C2PSA to strengthen inter-channel dependency modeling and improve feature discriminability; and a Focaler-IoU loss function to emphasize hard and small samples while reducing localization errors. In addition, we construct and annotate a dedicated tennis dataset containing 6,648 images across three categories—player, racquet, and ball—covering diverse scenes, camera angles, and lighting conditions. Experimental results show that YOLO-Net achieves 84.5% precision and 78.2% mAP@0.5 with only 2.58M parameters, outperforming the YOLO11n baseline by 2.5% in precision and 0.9% in mAP while maintaining real-time inference. These findings demonstrate that YOLO-Net is an efficient, accurate, and deployable solution for applications such as referee assistance, tactical analysis, and intelligent broadcasting in tennis competitions.

## Introduction

In modern competitive sports, tennis stands out for its agility, intensity, and technical sophistication, attracting widespread attention from athletes and enthusiasts alike. Recent studies further suggest that tennis may be regarded as one of the most “comprehensive” forms of exercise, capable of enhancing both physical and psychological capacities across multiple dimensions [1]. Within the professional arena, the precise detection of players and ball trajectories is of critical importance: it not only provides objective evidence for refereeing and statistical analysis but also supports performance evaluation, tactical planning, and intelligent event management. Comprehensive recognition of player positioning, movement trajectories, and cooperative patterns enables coaches and analysts to design training regimens tailored to individual athletes [2]. Moreover, real-time detection and tracking of the tennis ball allow for fine-grained measurement of velocity, spin, and landing points, thereby facilitating tactical interpretation and enriching broadcast quality [3].

In recent years, deep learning has emerged as a cornerstone of artificial intelligence, delivering remarkable advances across computer vision tasks. The integration of convolutional neural networks (CNN) with Transformer architectures has markedly improved the accuracy of image classification, object detection, and segmentation [4,5]. Beyond conventional vision tasks, recent AI models have also shown strong potential in complex dynamic-scene modeling, such as graph-based vehicle trajectory prediction in intelligent transportation systems [6]. In object detection, leading frameworks such as You Only Look Once (YOLO), Detection Transformer (DETR), Faster Region-based Convolutional Neural Network (Faster R-CNN), and Single Shot MultiBox Detector (SSD) have collectively accelerated progress in real-time recognition under complex conditions [7,8]. Yet, tennis-specific detection remains uniquely challenging. First, the ball is diminutive and moves at high velocity, creating sharp scale disparities relative to players and the net that hinder robust feature extraction [9]. Second, occlusions frequently arise among players, racquets, and the net, compounded by cluttered backgrounds that degrade detection accuracy. Third, variability in lighting and camera perspectives undermines robustness [10]. Although several tennis detection algorithms have been proposed, they often fail to balance accuracy, real-time performance, and adaptability under such constraints. These limitations underscore the urgent need for specialized approaches to enhance trajectory detection of both players and the ball in tennis scenarios.

To address these challenges, we propose YOLO-Net, a task-oriented lightweight detection framework built upon YOLO11n for tennis match scenarios. Rather than proposing a new general-purpose detection paradigm, YOLO-Net focuses on improving edge-aware feature representation, channel-level discriminability, and bounding-box regression for small and fast-moving tennis targets. YOLO-Net preserves real-time efficiency while improving the recognition of small objects, such as tennis balls, and structural targets, such as racquets and players. Our contributions are fourfold:

(1) We construct a tennis-specific object detection dataset comprising diverse court types, camera viewpoints, illumination conditions, and match scenarios, with fine-grained annotations of players, tennis racquets, and balls, enabling comprehensive evaluation in authentic tennis settings.
(2) We design a C3k-MSEIS module by introducing multi-scale edge enhancement and dual-domain feature selection into the YOLO11n backbone. This module strengthens the representation of complex boundaries and high-frequency details, thereby improving the detection of small and structural targets in tennis scenes.
(3) We integrate ECA channel attention after C2PSA to enhance channel-level feature recalibration with limited parameter overhead, improving feature discriminability and cross-scene robustness.
(4) We adopt Focaler-IoU for bounding-box regression to emphasize hard and extremely small samples and reduce localization drift. Together with C3k-MSEIS and ECA, it forms a lightweight, edge-enhanced YOLO11n-based framework for tennis object detection.

## Related work

### Object detection evolution

Object detection has undergone a rapid evolution from traditional hand-crafted feature methods to modern deep learning approaches. Early studies largely relied on sliding windows and manually designed descriptors such as Haar, HOG, and SIFT, which were typically combined with support vector machines or boosting classifiers to perform detection [11,12]. While these methods achieved limited success in constrained settings, they generally suffered from weak feature representation, sensitivity to illumination and pose variations, and high computational costs, making them unsuitable for deployment in complex real-world environments [13,14].

with the rapid progress of deep convolutional neural networks, learning-based detectors quickly became dominant. Representative two-stage approaches, such as the R-CNN family, integrate proposal mechanisms with convolutional features to substantially enhance detection accuracy [15]. Yet, depending on proposal networks often results in heavy computational costs, restricting their use in real-time applications. By contrast, one-stage detectors such as YOLO and SSD reformulated detection as a dense regression problem, striking a more favorable balance between accuracy and efficiency and thereby accelerating the adoption of real-time detection systems [16,17]. The YOLO family, in particular, has advanced rapidly: successive iterations have incorporated cross-stage partial connections, attention mechanisms, and anchor-free strategies. The latest version, YOLO11, further refines the trade-off between speed and precision, consolidating YOLO’s role as a leading paradigm in object detection [18].

More recently, Transformer-based architectures have been introduced into the field. DETR illustrates this paradigm shift by treating detection as sequence prediction, removing classical elements like proposals and non-maximum suppression, and offering an elegant end-to-end solution [19]. Despite its methodological simplicity and conceptual novelty, DETR faces practical challenges, including slow convergence and heavy computational demands, which hinder its suitability for real-time applications.

Overall, the trajectory of object detection research reflects a persistent effort to reconcile accuracy with efficiency, to strengthen robustness against small objects and complex environments, and to integrate multi-task learning with lightweight model design. These advances establish a solid foundation for the development of next-generation detection frameworks tailored to the unique challenges of tennis analysis.

### Tennis object detection

Early research on tennis detection primarily relied on handcrafted cues such as color, motion, and geometric features for target modeling. Archana et al. proposed a method based on logical operations and background subtraction, which, combined with connected-component analysis, enabled the joint recognition of tennis balls and players [20]. Rocha et al. integrated computer vision and morphological operations with machine learning to detect ball positions, court boundaries, and players, applying the results to assist refereeing decisions [21]. Saralıoğlu et al. employed image enhancement and adaptive filtering techniques to achieve accurate ball-impact detection even under low-resolution and low-frame-rate conditions [22]. Yu et al. developed a hybrid tracking algorithm that combined mean shift with color histograms to address appearance variations and complex motion in sports videos, achieving favorable accuracy and robustness [23]. Nonetheless, these approaches depended heavily on handcrafted features and threshold settings, rendering them vulnerable to variations in illumination, motion blur, and occlusion, while also lacking real-time performance and generalizability.

With the advent of deep learning, convolutional neural networks and end-to-end detectors have progressively supplanted traditional methods. Reno et al. proposed a CNN-based tennis detection framework capable of identifying the presence of a ball from single-frame images, achieving high accuracy in real match videos [24]. Wu et al. introduced an enhanced Mask R-CNN–based approach, in which loss function optimization improved detection performance; their method achieved consistently high recognition rates and demonstrated strong applicability [25]. Tian et al. designed an anchor-free detection strategy combined with data augmentation to effectively address the challenge of high-speed, small-object tennis detection [26]. Zhang et al. integrated YOLOv5 with TensorRT, boosting feature extraction precision while accelerating inference; their model achieved 94% mAP along with real-time performance, providing a reliable tool for match officiating [27]. More recently, Zhu et al. proposed an improved lightweight RTMDet-light architecture, incorporating large-kernel convolution, GhostNet, and ShuffleNet modules, which enabled efficient detection of high-speed tennis balls under complex, multi-shadow conditions [28]

### YOLO-based module combinations for small-object detection

Recent studies have increasingly improved YOLO-based detectors through combined designs involving attention mechanisms, multi-scale feature enhancement, lightweight convolutional modules, and optimized bounding-box regression losses. For example, CRL-YOLOv5 introduced CBAM and an RFB module to enhance local feature representation and contextual modeling for small-object detection [29]. YOLO-TLA added an additional small-object detection layer, a lightweight C3CrossConv module, and a global attention mechanism to improve fine-grained feature extraction while maintaining a compact model size [30]. LACF-YOLO integrated Triplet Attention, cross-scale feature fusion, lightweight convolutional structures, and Focal EIoU loss to improve small-object detection in remote-sensing scenarios [31]. LSOD-YOLOv8 further combined a lightweight module, a new loss function, and multi-scale information to enhance small-object detection under efficiency constraints [32]. Similarly, FDM-YOLO improved YOLOv8 by adding a high-resolution detection layer, Fast-C2f, dynamic upsampling, and EMA attention to balance detection accuracy and lightweight deployment [33]. More recently, AED-YOLO11 combined frequency-domain feature aggregation, efficient attention compression, dynamic upsampling, WIoU loss, and a P2 detection head to improve small-object recognition in complex scenes [34].

These studies indicate that module combinations are effective for improving YOLO-based small-object detection. However, most existing methods are designed for specific domains such as remote sensing, UAV imagery, traffic scenes, medical detection, or industrial defects. Their target scales, background distributions, motion characteristics, and deployment requirements differ from tennis match scenarios. Moreover, many methods rely on directly stacking attention modules, extra detection heads, or feature fusion blocks, which may increase computational cost and weaken the real-time advantage of lightweight YOLO detectors. Some approaches improve general feature saliency or multi-scale representation, but they do not explicitly enhance high-frequency edge cues that are critical for detecting tennis balls, racquet contours, and net-like structures.

## Methods

### Overall network structure

**Fig 1:**
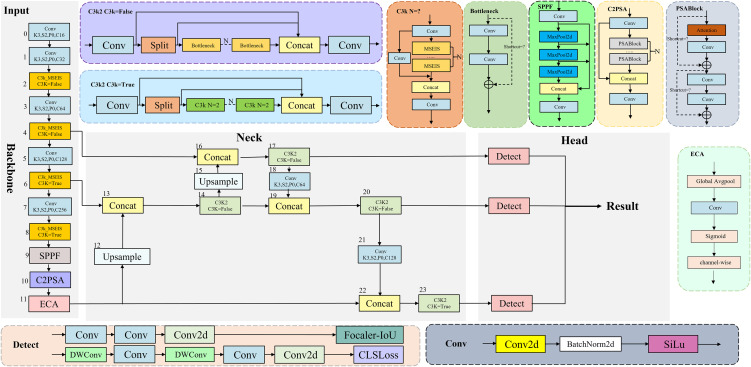
Network architecture diagram of YOLO-Net.

By improving and optimizing the baseline YOLO11 [35] framework, we propose a novel lightweight tennis detection network, YOLO-Net, whose overall architecture consists of four main components: the input module, backbone feature extraction network, feature aggregation and enhancement module, and detection head. As shown in Fig 1, the original YOLO11 structure provides efficient real-time detection but exhibits performance degradation in scenarios involving fast-moving, small-scale tennis balls with blurred edges. This is mainly due to limited multi-scale edge representation and insufficient robustness to occlusions and background interference.

To address these issues, YOLO-Net integrates a C3k_MSEIS module in the backbone to enhance multi-scale edge feature extraction and suppress irrelevant background information [36]. This improves the network’s ability to accurately capture fine-grained structural details of tennis balls, racquets, and net patterns. To further strengthen semantic representation, a lightweight ECA attention mechanism is incorporated to emphasize informative feature channels and improve detection robustness under varying lighting and occlusion conditions [37]. In addition, Focaler-IoU loss is employed for bounding box regression, enabling more accurate localization of small and partially occluded objects while accelerating convergence during training [38]. These architectural improvements allow YOLO-Net to maintain real-time inference speed while significantly enhancing detection precision for challenging tennis scenes.

### C3k_MSEIS module for multi-scale edge feature enhancement

In the YOLO11 backbone, the C3k2 module serves as a core feature extraction unit. By combining standard convolution with variable-kernel convolution or Bottleneck layers through a branched structure, it achieves a good balance between computational efficiency and feature representation, enabling the network to learn rich hierarchical semantic information with limited computational overhead. However, when applied to tennis object detection, this module exhibits inherent limitations in capturing fine-grained structural information. Specifically, the high-speed motion of tennis balls often leads to motion blur, their small size results in weak edge responses, and frequent occlusions from racquets and nets further increase background complexity. These factors make it difficult for the C3k2 module to preserve high-frequency details and clear object boundaries, ultimately causing degraded localization and classification accuracy. In addition, the feature fusion process of C3k2 lacks explicit modeling of edge information, making it prone to confusing tennis balls with visually similar background textures. These issues highlight the necessity of incorporating explicit edge enhancement and multi-scale feature selection mechanisms into the backbone to better strengthen the representation of subtle, high-frequency features.

To overcome these limitations, we introduce the Multi-Scale Edge Information Select (MSEIS) mechanism into the C3k2 design, yielding the improved C3k_MSEIS module. Inspired by the Dual-Domain Selection Mechanism (DSM) widely applied in image restoration, this module performs feature selection simultaneously in the spatial and frequency domains, thereby enhancing the modeling of edge structures and high-frequency details. The overall design consists of two stages: edge enhancement and multi-scale selection. From an implementation perspective, C3k_MSEIS follows a progressive feature refinement process. Given an input feature map, the module first generates multi-scale contextual representations through adaptive average pooling with different output sizes, where k = {3, 6, 9, 12}. Each pooled feature is then upsampled to the original spatial resolution and passed through the EdgeEnhancer branch to emphasize high-frequency boundary cues. The EdgeEnhancer suppresses smooth background responses while retaining residual edge information, which is particularly useful for small and blurred tennis balls, racquet contours, and net-like structures. The enhanced multi-scale features are concatenated with the local convolutional branch and then processed by the dual-domain selection mechanism. In this way, C3k_MSEIS combines local texture preservation, multi-scale contextual aggregation, and edge-aware feature selection within a lightweight backbone module.

**Fig 2:**
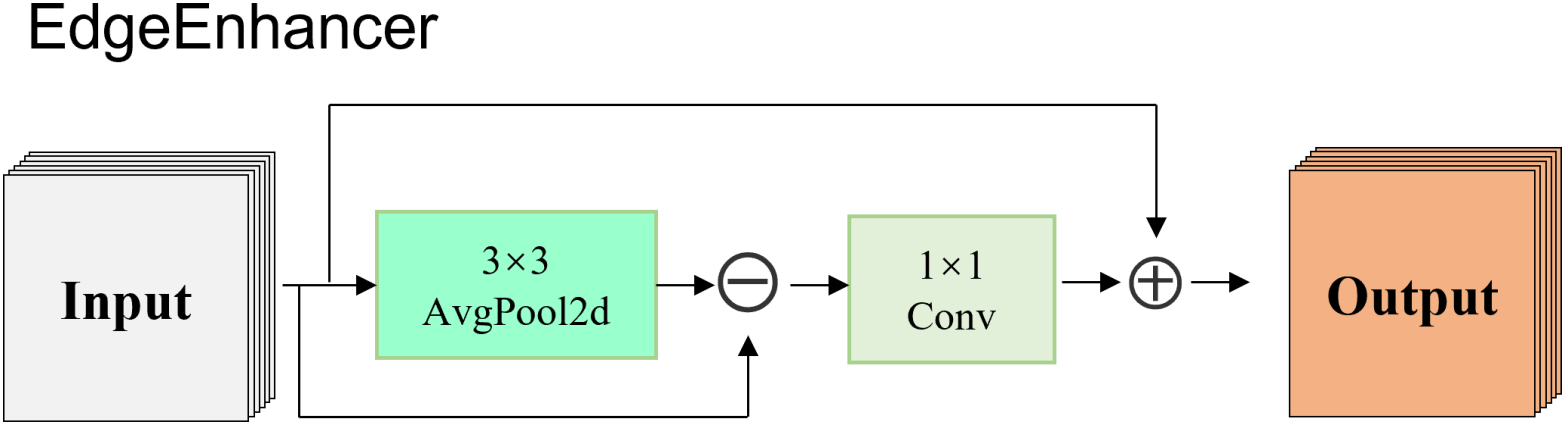
EdgeEnhancer module structure diagram.

To extract salient edge information, we design a lightweight EdgeEnhancer module, as illustrated in Fig 2. Its core strategy constructs a residual enhancement branch through average pooling and convolutional mapping, enabling the separation of edge details from the original feature maps:

To extract salient edge information, we design a lightweight EdgeEnhancer module, as illustrated in Fig 2. Its core strategy constructs a residual enhancement branch through average pooling and convolutional mapping, enabling the separation of edge details from the original feature maps:


$$\begin{array}{c} X_{edge}=X_{in}\;-\;{\text{Pool}(X}_{in}) \end{array}$$ (1)

Pool(·) denotes a 3 × 3 average pooling operation applied to smooth the input features. The resulting map is then passed through a 1 × 1 convolution followed by a Sigmoid activation to generate the corresponding edge weights:


$$\begin{array}{c} W\;=\;\sigma ({Conv}_{\text{1}x\text{1}}(X_{edge})) \end{array}$$ (2)

The enhanced features are ultimately derived.


$$\begin{array}{c} X_{out}\;=\;X_{in}\;+\;W\;⊙\;X_{edge} \end{array}$$ (3)

In this way, the model is able to highlight boundary contours and fine-detail regions, thereby enhancing its sensitivity to edge features that are critical for detecting small objects and handling complex scenes.

**Fig 3:**
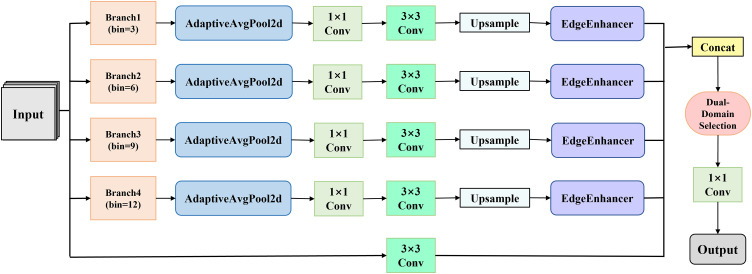
MSEIS module structure diagram.


On this basis, we introduce a multi-branch structure that performs adaptive down-sampling and convolutional mapping on the input feature as illustrated in Fig 3. Specifically, for each scale *k*∈{3,6,9,12}, an adaptive average pooling operation is first applied to obtain a feature representation at the corresponding resolution, denoted as ${\;F}^{\left(k\right)}$:

$$\begin{array}{c} {\;F}^{\left(k\right)}{=Conv}_{\text{3}\times \text{3}}({Conv}_{\text{3}\times \text{3}}({Pool}_{k}(F)) \end{array}$$ (4)

${Pool}_{k}\;$denotes an average pooling operation with an output size of *k × k* The resulting feature maps are then upsampled back to the original resolution and passed through the EdgeEnhancer module for high-frequency refinement, producing the enhanced features $\overset{\left(k\right)}{\underset{edge}{F}}$.

Finally, the multi-scale enhanced features are concatenated and fed into the Dual-Domain Selection Mechanism where feature screening is performed simultaneously in both the spatial and frequency domains:


$$\begin{array}{c} F_{mseis}=\;DSM(Concat([F_{local},\overset{\left(\text{3}\right)}{\underset{edge}{F}},\overset{\left(\text{6}\right)}{\underset{edge}{F}},\overset{\left(\text{9}\right)}{\underset{edge}{F}},\overset{\left(\text{1}\text{2}\right)}{\underset{edge}{F}}])) \end{array}$$ (5)

$F_{local}\;$denotes the local convolutional branch. The introduction of DSM enables the adaptive suppression of redundant noise while highlighting critical edge regions, thereby yielding the final discriminative features.

### Efficient channel attention

**Fig 4:**
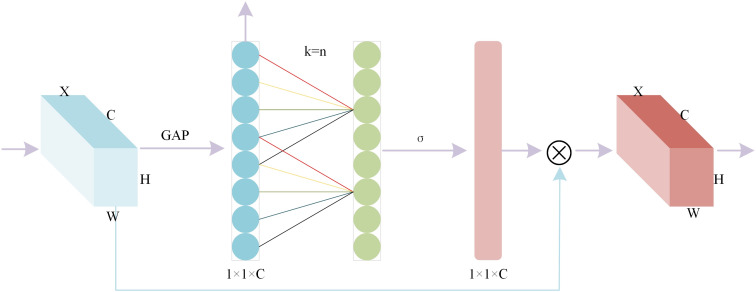
ECA structure diagram.

In the YOLO11 detection framework, the C2PSA module enables hierarchical modeling of multi-scale features, but its capacity for inter-channel information interaction remains limited. To further enhance the discriminative power of feature representations, we incorporate the ECA mechanism into the C2PSA module. Unlike SE attention, ECA employs one-dimensional convolution to model local channel interactions, thereby capturing cross-channel dependencies without the need for fully connected layers. Moreover, it adaptively determines the kernel size according to the number of channels, achieving channel recalibration with minimal parameter overhead. The overall structure is illustrated in Fig 4.

Embedding ECA after C2PSA allows the two modules to complement one another: while C2PSA strengthens multi-scale feature aggregation, ECA refines channel-level discriminability. Through this mechanism, ECA adaptively assigns weights across channels, suppressing redundant or irrelevant responses while highlighting task-relevant information. Specifically, features processed by C2PSA undergo a secondary refinement when passed through ECA, further accentuating discriminative channel characteristics. This not only ensures effective separation of tennis balls, players, and complex backgrounds but also improves the model’s adaptability and robustness across diverse postures and scene conditions.

### Focaler-IoU

As a core task in computer vision, the performance of object detection is heavily influenced by the design of its loss functions. Traditional detection losses primarily focus on geometric metrics for bounding-box regression—such as the distance between predicted and ground-truth boxes, intersection-over-union (IoU), and aspect ratios. Representative examples include GIoU, CIoU, and EIoU [33]. However, these methods often fail to account for angular deviations between predicted and ground-truth boxes, which may lead to localization drift during training. Such drift not only slows convergence but also reduces training efficiency and ultimately compromises detection accuracy.

To address this issue, Hao Zhang et al. [32] proposed the Focaler-IoU loss function, which reconstructs the original IoU loss through a linear interval mapping. This design adaptively emphasizes the imbalance between easy and hard samples. In object detection, sample difficulty varies substantially: conventional objects are relatively easy to detect, whereas extremely small objects—due to blurred boundaries and scale limitations—typically fall into the “hard” category. Focaler-IoU is specifically designed to strengthen the modeling of such challenging samples, thereby enhancing overall detection performance across diverse scenarios.

By employing linear interval mapping to rebuild the IoU loss, the method allows the regression process to better focus on different categories of samples and tasks, ultimately improving boundary regression. The formulation is given as follows:


$$\begin{array}{c} \begin{array}{c} {IoU}^{f}=\left\{ \begin{array}{c} @c\text{0},\;IoU<T_{l}\\ \frac{IoU-T_{l}}{T_{h}-T_{l}},\;T_{l}\ll IoU\ll T_{h}\\ \text{1},\;IoU>T_{h}\; \end{array}\right.\; \end{array} \end{array}$$ (6)

${IoU}^{f}\;$denotes the reconstructed Focaler-IoU, while IoU represents the original IoU value, with [$T_{l}$, $T_{h}$]∈[0,1]. By adjusting the values of $T_{l}$ and $T_{h}$, ${IoU}^{f}$ can be directed to focus on different categories of regression samples. The corresponding loss is defined as follows:


$$\begin{array}{c} \begin{array}{c} L_{F-IoU}=\text{1}-{IoU}^{f} \end{array} \end{array}$$ (7)

The Focaler-IoU loss can be integrated into existing IoU-based bounding box regression losses, yielding the following formulation for $L_{F-\text{C}IoU}$:


$$\begin{array}{c} \begin{array}{c} L_{F-\text{C}IoU}=L_{CIoU}+IoU-{IoU}^{f} \end{array} \end{array}$$ (8)

In the proposed YOLO-Net model, incorporating Focaler-IoU enhances localization accuracy and generalization capability. Focaler-IoU leverages the comprehensive optimization of CIoU with respect to center-point distance, overlap area, and aspect ratio, while introducing a dynamic linear interval mapping over [$T_{l}$, $T_{h}$] to provide an adaptive focus mechanism for samples of varying difficulty. For low-IoU, hard-to-detect targets—such as small or low-quality objects—reducing the value of $T_{l}$ increases gradient contributions, thereby mitigating missed detections. Conversely, for high-IoU, easy-to-detect targets of high quality, raising the value of $T_{h}$ refines localization details and reduces redundant regression.

## Results

### Datasets

This study is based on a tennis object detection dataset specifically constructed for the present research. Using web-crawling techniques, we collected publicly available tennis match videos from major streaming platforms and extracted representative frames to generate a static image dataset. The resulting dataset contains 6,648 images with a resolution of 1280 × 1280 pixels, covering a wide range of tennis match scenarios, including different court types, lighting conditions, camera viewpoints, player poses, and background complexities. All collected videos were publicly available on their original platforms, and the data collection and analysis procedures complied with the applicable terms and conditions of these data sources. The dataset includes three object categories: player, tennis racquet, and ball. In total, 21,144 object instances were annotated across the 6,648 images. Specifically, the player category appears in 6,434 images and contains 11,414 annotated instances; the tennis racquet category appears in 4,708 images and contains 5,754 annotated instances; and the ball category appears in 3,342 images and contains 3,976 annotated instances. These statistics indicate that the dataset contains both large structural targets, such as players, and small high-speed targets, such as tennis balls, making it suitable for evaluating object detection performance in challenging tennis match scenarios.

During dataset construction, particular attention was paid to scene diversity and annotation reliability. The collected images cover variations in court surface, illumination, camera perspective, player posture, background interference, long-distance views, and small tennis-ball targets, all of which increase the difficulty of object detection. Each image was manually labeled according to standard object detection annotation criteria, and bounding-box annotations were generated for all visible instances of the three target categories. To ensure annotation consistency and quality, the annotations were subjected to multiple rounds of checking and correction. Therefore, the constructed dataset provides a reliable benchmark for training and evaluating tennis object detection models.

### Experimental platform and parameter settings

The proposed algorithm was implemented using the PyTorch framework (version 1.8) with Python 3.8. Experiments were conducted on a Linux system (Ubuntu 22.04) equipped with an Intel Xeon E5-2650 v3 CPU (2.30 GHz), 64 GB of RAM, and a NVIDIA V100 GPU with 32 GB of memory. The environment was further supported by CUDA 11.8 for parallel computation and cuDNN 8.2 for deep neural network acceleration.

For training, images were resized to 640 × 640 and processed in batches of 128 samples over 200 epochs. Optimization was performed using stochastic gradient descent (SGD) with an initial learning rate of 0.01, a momentum coefficient of 0.937, and an L2 regularization factor of 0.005. This configuration, combined with dynamic learning-rate scheduling, momentum acceleration, and weight decay, effectively controlled model complexity and improved convergence during training. The detailed hyperparameter settings are summarized in Table 1. To ensure reproducibility and fair comparison, the original YOLO11n default settings were retained for all parameters not explicitly listed in Table 1, including data augmentation, loss weights, confidence threshold, NMS configuration, and other training and inference settings. No additional manual tuning was applied to different comparison models.

**Table 1:** Training configuration and hyperparameter settings.

Training Configuration	Value
Epochs	200
Optimizer	SGD
Momentum	0.937
Batch size	128
Weight decay	5 × 10 ^− 4^
Warm-up epochs	3
Warm-up momentum	0.8
Warm-up bias learning rate	0.0
Initial learning rate	10 ^− 2^
Final learning rate	10 ^− 4^
Learning rate schedule	Linear decay

To comprehensively verify the effectiveness of the proposed modules integrated into the YOLO11n framework, a series of ablation and model comparison experiments were conducted. All experiments were performed on the same dataset and under identical training configurations to ensure fair evaluation. Four standard metrics were adopted to quantitatively assess the detection performance: Parameters (Params), Precision (P), Recall (R), and mean Average Precision at IoU threshold 0.5 (mAP@0.5).

The Precision and Recall are defined as


$$\begin{array}{c} P=\frac{TP}{TP+FP} \end{array}$$ (9)

$$\begin{array}{c} R=\frac{TP}{TP+FN} \end{array}$$ (10)

where TP denotes correctly detected objects, FP represents incorrect detections, and FN indicates missed detections.

The mean Average Precision at loU threshold 0.5, denoted as mAP@0.5, is computed as the mean of the average precision values over all categories, with loU ≥ 0.5 considered as a correct detection:


$$\begin{array}{c} mAP=\frac{\text{1}}{N}\sum_{i=\text{1}}^{N}{AP}_{i} \end{array}$$ (11)

where *N* is the number of object categories and ${AP}_{i}$ is the average precision for the *i*-th class.

### Comparative experiments

To validate the superiority of the proposed tennis object detection model, we conducted a systematic and comprehensive set of comparison experiments on the self-constructed tennis dataset. Specifically, several representative lightweight YOLO-series object detection models were selected for comparison, including YOLOv5n, YOLOv8n, YOLOv10n, YOLO11n, YOLO12n, and YOLO26n. The results, summarized in Table 2, clearly demonstrate the outstanding performance of the proposed YOLO-Net in tennis detection tasks, thereby further substantiating its significant advantages in this domain.

**Table 2:** Comparison of YOLO-Net with lightweight YOLO-series detection models.

Model	Parameter	Precision	Recall	mAP@.5
YOLOv5n	2.18M	83.4%	72.8%	77.0%
YOLOv8n	2.69M	81.5%	73.8%	77.3%
YOLOv10n	2.70M	81.1%	72.4%	77.7%
YOLO11n	2.62M	82.0%	73.2%	77.3%
YOLO12n	2.60M	80.4%	73.2%	77.3%
YOLO26n	2.40M	83.6%	73.1%	77.9%
YOLO-Net	2.58M	84.5%	73.0%	78.2%

**Fig 5:**
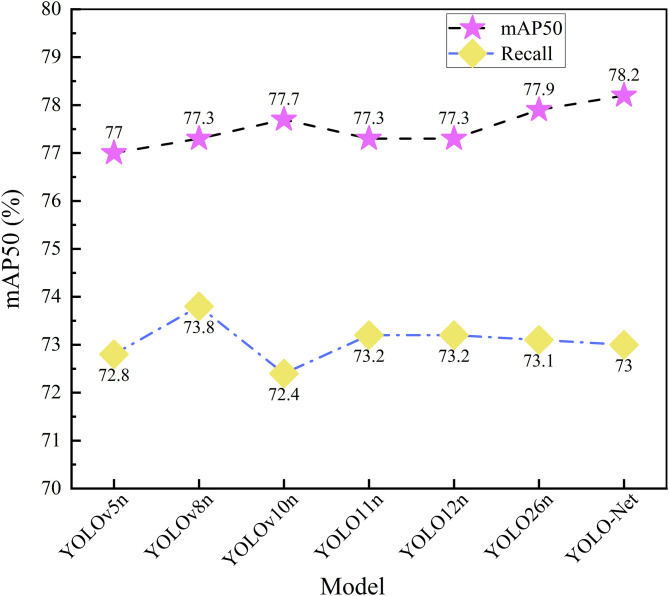
Performance comparison across different models.

As shown in Table 2 and Fig 5, YOLO-Net achieved the highest Precision of 84.5% and the highest mAP@0.5 of 78.2% among the evaluated lightweight YOLO-series models. Compared with YOLO11n, YOLO-Net improved Precision from 82.0% to 84.5% and mAP@0.5 from 77.3% to 78.2%, while maintaining a comparable parameter count of 2.58M. These results suggest that YOLO-Net provides a favorable balance between detection performance and model size for small-object detection in tennis match scenarios.

In addition to the quantitative results, we further examined representative validation cases to assess the detection behavior of YOLO-Net under challenging tennis match scenarios. Compared with YOLO11, YOLO-Net showed more stable recognition of small tennis-ball targets, stronger suppression of false detections caused by background interference or racquet-like structures, and improved robustness under fast ball-motion conditions. These observations are consistent with the quantitative results in Table 2 and further indicate that YOLO-Net improves feature discriminability and localization stability for small and visually ambiguous tennis objects.

### Ablation studies

In this section, we conduct systematic ablation experiments to analyze the contribution of each proposed component in YOLO-Net to the overall detection performance. By decomposing the network into its core innovative modules—C3k_MSEIS, ECA, and Focaler-IoU—we aim to intuitively illustrate how each module affects the model’s accuracy, efficiency, and robustness. The experiments are designed to examine the specific functional role of each component and to quantify its contribution to performance improvements.

The ablation experiments are based on the YOLO11n backbone using the same dataset and training configuration to ensure consistency and fairness. We systematically evaluate the effects of the three modules on key performance indicators, including the number of model parameters, Precision, Recall, and mAP@0.5. A comprehensive comparison is carried out across multiple dimensions, providing a clear understanding of how each module contributes to overall detection capability.

In these experiments, Model A represents the baseline YOLO11n without any modifications. Model B adds the C3k_MSEIS module to enhance the extraction of multi-scale edge features and strengthen fine-grained boundary representation. Model C builds upon Model B by incorporating the ECA channel attention mechanism, aiming to improve inter-channel dependency modeling and robustness to background noise. Model D further introduces the Focaler-IoU loss function to optimize bounding-box regression, particularly for hard samples and extremely small targets. The detailed experimental results are presented in Table 3.

**Table 3:** Ablation experiments of the models.

Model	C3k_MSEIS	ECA	Focaler-IoU	Parameter	Precision	Recall	mAP@.5
A				2.62M	82.0%	73.2%	77.3%
B	✓			2.58M	81.6%	71.8%	78.0%
C	✓	✓		2.58M	83.5%	73.1%	78.0%
D	✓	✓	✓	2.58M	84.5%	73.0%	78.2%

**Fig 6:**
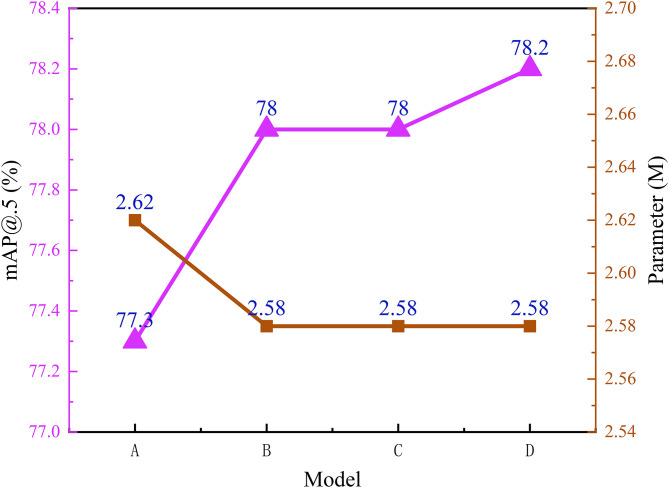
Ablation study of different modules.

As shown in Fig 6, the experimental results demonstrate that the proposed modules significantly improve model performance while preserving lightweight characteristics. Specifically, the C3k_MSEIS module effectively enhances edge feature modeling in complex scenarios, leading to a noticeable increase in mAP@0.5 compared with the baseline. Building upon this, the integration of the ECA channel attention mechanism further strengthens inter-feature dependencies, resulting in a marked improvement in Precision. Finally, the incorporation of the Focaler-IoU loss alleviates the imbalance encountered during training on extremely small or hard samples, ultimately achieving higher detection accuracy and robustness.

The ablation study confirms the complementary and synergistic effects of the three modules within the YOLO11n framework: MSEIS refines edge features, ECA reinforces channel-level interactions, and Focaler-IoU enhances modeling of small objects. Their combined use not only boosts overall detection performance but also achieves an optimal balance among accuracy, parameter size, and computational cost. These results strongly validate the effectiveness and practical potential of the proposed improvements in lightweight object detection tasks.

### Comparative analysis of attention mechanisms

**Fig 7:**
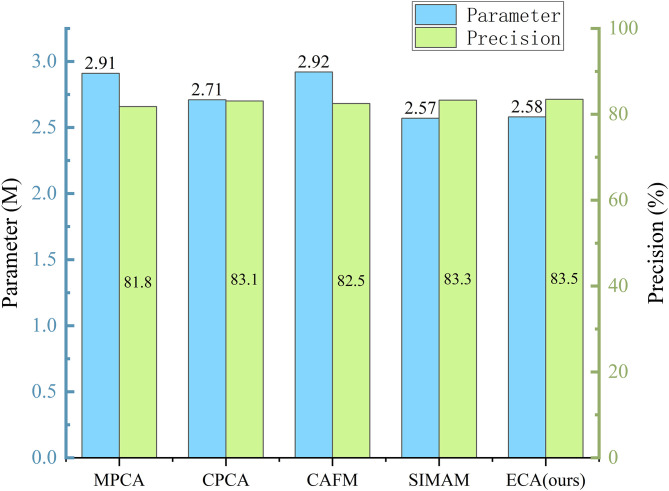
Comparative experiments of different attention mechanisms.

To further evaluate the modeling capability and accuracy differences among various attention mechanisms in tennis object detection, we conducted comparative experiments by incorporating several mainstream modules into the C3k_MSEIS framework. The tested modules include MPCA, CPCA, CAFM, SIMAM, and ECA. The experimental results are summarized in Table 4 and illustrated in Fig 7.

**Table 4:** Comparative experiments of different attention mechanisms.

Model	Parameter	Precision	Recall	mAP@.5
YOLO11n-c3k_MSEIS_MPCA	2.91M	81.8%	72.1%	76.7%
YOLO11n-c3k_MSEIS_CPCA	2.71M	83.1%	72.8%	77.0%
YOLO11n-c3k_MSEIS_CAFM	2.92M	82.5%	72.3%	77.2%
YOLO11n-c3k_MSEIS_SIMAM	2.57M	83.3%	71.8%	77.7%
YOLO11n-c3k_MSEIS_ECA	2.58M	83.5%	73.1%	78.0%

Overall, all attention mechanisms improved the discriminative capacity of the model to some extent, but their performance varied considerably. CPCA and SIMAM demonstrated relatively balanced outcomes in terms of Precision and Recall, reaching 83.1%/72.8% and 83.3%/71.8%, respectively, indicating a degree of robustness in target recognition under background interference. CAFM yielded moderate improvements in Precision and Recall, but its larger parameter size limited computational efficiency. By contrast, the ECA module achieved the best trade-off: with the smallest parameter count (2.58M), it delivered the highest detection Precision (83.5%) and Recall (73.1%), and attained an mAP@0.5 of 78%. These results highlight the superior balance of performance and efficiency achieved by ECA, making it a more suitable choice for lightweight and accurate detection in tennis scenarios.

### Comparison of different IoU loss functions

To comprehensively assess the applicability of different bounding-box regression loss functions in the improved YOLO11n model, we conducted comparative experiments under a unified dataset and training configuration. The tested loss functions include common variants such as CIoU, EIoU, DIoU, GIoU, bbox_mpdIoU, and inner_CIoU, which were compared against the Focaler-IoU introduced in this study. The experimental results are summarized in Table 5.

**Table 5:** Comparative experiments of different IoU loss functions.

Model	Parameter	Precision	Recall	mAP@.5
CIoU	2.58M	83.5%	73.1%	78.0%
EIoU	2.58M	81.6%	73.1%	77.8%
DIoU	2.58M	82.3%	73.1%	77.8%
GIoU	2.58M	82.1%	72.8%	77.2%
bbox_mpdIoU	2.58M	83.4%	72.7%	77.5%
inner_CIoU	2.58M	80.9%	73.6%	76.7%
Focaler-IoU	2.58M	84.5%	73.0%	78.2%

From the results, traditional loss functions such as CIoU, DIoU, and GIoU exhibit a degree of stability in complex scenarios. Among them, CIoU achieves performance comparable to the baseline but shows certain shortcomings in Recall. EIoU and inner_CIoU yield slight improvements in Recall, yet their mAP fluctuates considerably, suggesting limited adaptability to small objects and occlusion cases. In contrast, the proposed Focaler-IoU achieves the best outcomes in both Precision and mAP@0.5, with Precision reaching 84.5% and mAP attaining 78.2%, substantially outperforming other methods. These results indicate that Focaler-IoU can more effectively focus on hard samples and extremely small targets, thereby enhancing the overall detection performance of the model.

## Discussion

This study proposes YOLO-Net, a lightweight and enhanced object detection framework designed specifically for tennis scenarios. YOLO-Net is not intended as a fundamentally new general-purpose detection architecture; instead, it represents a task-oriented adaptation of YOLO11n for tennis small-object detection. By combining multi-scale edge feature enhancement, lightweight channel attention, and improved bounding-box regression, the model improves detection performance for small and fast-moving targets in complex tennis environments. The C3k_MSEIS module explicitly introduces edge-aware feature selection in the backbone, enhancing the network’s sensitivity to high-frequency details such as tennis ball edges, racquet meshes, and net structures. The ECA attention mechanism strengthens the model’s discriminative power under varying lighting and background conditions without introducing considerable computational overhead. Meanwhile, the Focaler-IoU loss improves the localization of extremely small and occluded targets and stabilizes the training process. Although ECA attention and Focaler-IoU have been explored in previous studies [37–39], their integration in YOLO-Net is task-oriented rather than purely additive. In this work, C3k_MSEIS provides edge-aware multi-scale feature enhancement in the backbone, ECA refines channel-level discriminability, and Focaler-IoU improves bounding-box regression for small and hard samples. This combination is designed specifically for tennis match scenarios characterized by small fast-moving balls, racquet occlusion, motion blur, and complex backgrounds.

Despite these improvements, several limitations remain. First, although the current experiments compare YOLO-Net with several representative lightweight YOLO-series models and include ablation studies, attention-mechanism comparisons, and IoU-loss comparisons, the experimental evaluation can be further expanded. Future work will include more diverse non-YOLO and small-object-oriented detection baselines, as well as additional evaluation metrics such as mAP@0.5:0.95, FPS, inference latency, GFLOPs, and class-wise AP to provide a more comprehensive assessment of accuracy, efficiency, and real-time deployment performance. Second, the scale and diversity of the current dataset are still limited, which may affect the model’s generalization to unseen match conditions. In extreme scenarios involving severe motion blur or uncommon camera angles, performance degradation may still occur. Moreover, temporal information across consecutive video frames has not yet been fully exploited. Future work may explore temporal modeling, lightweight Transformer-based modules, and advanced data augmentation strategies to further enhance YOLO-Net’s robustness and generalization in challenging real-world tennis environments.

## Conclusions

In this study, we proposed YOLO-Net, an improved lightweight detection framework tailored for tennis applications. By introducing the C3k_MSEIS module for multi-scale edge enhancement, the ECA channel attention mechanism, and the Focaler-IoU loss for bounding box regression, the model effectively addresses the challenges of small target size, rapid motion, blurred edges, and frequent occlusions. Experimental results demonstrate that YOLO-Net achieves higher precision and mAP@0.5 compared with multiple baseline YOLO models, while maintaining a lightweight parameter size and real-time inference speed. These findings indicate that YOLO-Net provides an effective and stable solution for intelligent tennis analysis and has strong potential for extension to other small-object detection tasks in complex scenarios.
