# Supplementary material for: YOLO-Net: A lightweight edge-enhanced detection model for small-object recognition in tennis match scenarios
Source: PLoS One. 2026 Jul 14;21(7):e0335558. doi: 10.1371/journal.pone.0335558 (PMC13367711; doi:10.1371/journal.pone.0335558)
Supplement: Attachment — Submitted filename: Response to Reviewers.docx [file pone.0335558.s002.docx]

**Summary of Changes**

Dear Editors and Reviewers,

We would like to sincerely thank you for your careful review and valuable comments on our manuscript entitled “YOLO-Net: A lightweight edge-enhanced detection model for small-object recognition in tennis match scenarios.” We have carefully revised and refined the manuscript in accordance with the journal requirements and the suggestions provided by the reviewers. We believe that these constructive comments have substantially improved the clarity, methodological rigor, reproducibility, and technical presentation of the revised version.

Thank you again for your time and effort in reviewing our work. We sincerely appreciate your consideration.

Best regards,

Liangquan Jia

On behalf of all authors

May 29, 2026

**Editor and Journal Requirements:**

**(1) Please ensure that your manuscript meets PLOS ONE style requirements, including those for file naming.**

Answer: Thank you for this important requirement. We have carefully checked the revised manuscript against the PLOS ONE formatting guidance, including the title page, main text structure, figure/table placement, references, file naming, line numbering, and page numbering. The revised submission package has also been prepared according to the requested file labels: “Response to Reviewers,” “Revised Manuscript with Track Changes,” and “Manuscript.”

*(Modifications in the manuscript: Entire manuscript and submission files.)*

---------------------------------------------------------------------------------------------------------------------------------

**(2) In the Methods section, please include additional information about the dataset and specify whether the collection and analysis method complied with the terms and conditions for the source of the data.**

Answer: Thank you for this important comment. We have revised the Datasets subsection in the Methods section by adding further information on the dataset construction process, target categories, annotation statistics, and quality control procedure. The revised manuscript now states that the dataset contains 6,648 images with a resolution of 1280 × 1280 pixels and 21,144 annotated object instances across three categories: player, tennis racquet, and ball.

In addition, we have added a sentence clarifying that the collected videos were publicly available on their original platforms and that the data collection and analysis procedures complied with the applicable terms and conditions of these data sources.

*(Modifications in the manuscript: Datasets subsection, pages 15–16, lines 306–332.)*

---------------------------------------------------------------------------------------------------------------------------------

**(3) Please review PLOS ONE code sharing guidelines and ensure that author-generated code is shared in a way that facilitates reproducibility and reuse.**

Answer: Thank you for this important reminder. We have carefully reviewed the PLOS ONE guidelines on materials, software, and code sharing, and we fully understand the importance of reproducibility and reuse. At present, the complete source code is still being used for our ongoing follow-up research and further methodological development, and therefore it is not yet suitable for unrestricted public release.To support reproducibility, the manuscript provides detailed descriptions of the YOLO-Net network architecture, module design, training configuration, hyperparameter settings, experimental platform, evaluation metrics, and comparison protocols. In particular, the revised manuscript reports the model structure, the design of C3k_MSEIS, ECA, and Focaler-IoU, as well as the training settings including input resolution, optimizer, batch size, learning rate, momentum, weight decay, warm-up strategy, and number of epochs. We believe that these details provide sufficient information for readers to understand and reproduce the main methodological workflow and experimental findings of this study.

---------------------------------------------------------------------------------------------------------------------------------

**(4) Thank you for stating in your Funding Statement: “This work was supported by the MOE (Ministry of Education in China) Liberal Arts and Social Sciences Foundation under Grant 21YJCZH191.” Please provide an amended statement that declares all the funding or sources of support received during this study. Please also include the statement “There was no additional external funding received for this study.” in your updated Funding Statement.**

Answer: Thank you for this reminder. We have amended the Funding Statement to declare the funding support received during this study and have included the required statement specified by the journal. The updated Funding Statement is as follows: “This work was supported by the MOE (Ministry of Education in China) Liberal Arts and Social Sciences Foundation under Grant 21YJCZH191. There was no additional external funding received for this study.” We have also included this amended Funding Statement in the cover letter.

*(Modifications in the cover letter and Funding Statement.)*

---------------------------------------------------------------------------------------------------------------------------------

**(5) Thank you for stating the following financial disclosure: “This work was supported by the MOE (Ministry of Education in China) Liberal Arts and Social Sciences Foundation under Grant 21YJCZH191 “Please state what role the funders took in the study. If the funders had no role, please state: "The funders had no role in study design, data collection and analysis, decision to publish, or preparation of the manuscript." If this statement is not correct you must amend it as needed.**

Answer: Thank you for this reminder. The funder refers to the funding agency that supported this study, namely the MOE (Ministry of Education in China) Liberal Arts and Social Sciences Foundation under Grant 21YJCZH191. The funders had no role in the study design, data collection and analysis, decision to publish, or preparation of the manuscript. We have included this amended Role of Funder statement in the cover letter as requested.

*(Modifications in the cover letter and Funding Statement.)*

---------------------------------------------------------------------------------------------------------------------------------

**(6) We note that you have indicated that there are restrictions to data sharing for this study. PLOS only allows data to be available upon request if there are legal or ethical restrictions on sharing data publicly. Before we proceed with your manuscript, please address the following prompts.**

**(7) The current data access contact point is a co-author. Please revise the data statement to provide a non-author institutional point of contact and include contact information for the third-party organization and the full citation of where the data can be found.**

Answer: Thank you for these important comments. We understand the PLOS ONE data availability policy and have revised our Data Availability statement accordingly. In the original submission, we indicated that the data were not publicly available for proprietary reasons and provided a contact point for data access. After further review, we have removed this access restriction and made the validation dataset used to support the evaluation results publicly available through a stable public repository.The validation dataset and corresponding annotation files have been deposited in Kaggle and are publicly available at: https://www.kaggle.com/datasets/taowang1123/yolo-net. Therefore, the previous “available upon request” statement and the author-based contact point are no longer applicable. The revised Data Availability statement now clearly states that the validation data and annotations are publicly available from the Kaggle repository.

*(Modifications in the manuscript: Data Availability statement, pages 26, lines 509–512.)*

---------------------------------------------------------------------------------------------------------------------------------

**(8) PLOS requires an ORCID iD for the corresponding author in Editorial Manager on papers submitted after December 6th, 2016. Please ensure that you have an ORCID iD and that it is validated in Editorial Manager. To do this, go to ‘Update my Information’ (in the upper left-hand corner of the main menu), and click on the Fetch/Validate link next to the ORCID field. This will take you to the ORCID site and allow you to create a new iD or authenticate a pre-existing iD in Editorial Manager.**

Answer: Thank you for this reminder. We will ensure that the corresponding author’s ORCID iD is validated in Editorial Manager according to the journal’s instructions before submitting the revised manuscript.

---------------------------------------------------------------------------------------------------------------------------------

**(9) Please ensure that you refer to Figure 8 in your text as, if accepted, production will need this reference to link the reader to the figure.**

Answer: Thank you for this reminder. We have revised the manuscript to explicitly refer to Fig. 8 in the main text. The revised sentence now reads: “As shown in Fig. 8, the experimental results demonstrate that the proposed modules significantly improve model performance while preserving lightweight characteristics.” This ensures that the figure is properly linked to the corresponding discussion in the text.

*(Modifications in the manuscript: Ablation Studies subsection, pages 19, lines 409–410.)*

---------------------------------------------------------------------------------------------------------------------------------

**(10) If the reviewer comments include a recommendation to cite specific previously published works, please review and evaluate these publications to determine whether they are relevant and should be cited. There is no requirement to cite these works unless the editor has indicated otherwise.**

Answer: Thank you for this reminder. We have carefully reviewed and evaluated the publications recommended in the reviewer comments. We understand that these references are not mandatory and should be cited only when they are directly relevant to the manuscript. After evaluation, we found that some of the recommended publications concern broader recent developments in artificial intelligence, while the present study focuses specifically on lightweight YOLO-based object detection for tennis match scenarios. Therefore, we have considered these publications carefully and cited only those that are directly relevant to our research scope and methodological discussion.

**Reviewer 1:**

The reviewer recognized the practical value of small-object detection in tennis match scenarios and the coherent improvements made to YOLO11n. The following issues were raised:

**(1) Clarify the exact novelty and contribution. Right now, the work looks like a well-engineered combination rather than a fundamentally new detection architecture.**

Answer: Answer: Thank you for this insightful comment. We agree that the novelty and contribution of the proposed method should be stated more clearly. We did not intend to claim that YOLO-Net is a fundamentally new general-purpose detection architecture. Rather, the contribution of this work lies in a task-oriented lightweight adaptation of YOLO11n for small-object recognition in tennis match scenarios.To avoid potential misunderstanding, we have revised the wording in the Abstract, Introduction, Overall Network Structure, and Discussion sections. In particular, “the framework introduces three innovations” has been revised to “the framework integrates three task-oriented improvements,” and “a novel lightweight tennis detection network” has been revised to “a task-oriented lightweight tennis detection framework.” We have also clarified that the main structural contribution is the C3k_MSEIS module, which introduces multi-scale edge enhancement and dual-domain feature selection into the YOLO11n backbone. ECA attention and Focaler-IoU are integrated as complementary components to improve feature discriminability and bounding-box regression for small and hard samples. These revisions better define the novelty of YOLO-Net as an edge-enhanced, tennis-specific improvement of YOLO11n rather than a completely new detection paradigm.

*(Modifications in the manuscript: Abstract, Introduction, Overall Network Structure, and Discussion sections, pages 2, 4, 10, 22 and 25)*

---------------------------------------------------------------------------------------------------------------------------------

**(2) Experimental evaluation needs to be strengthened. Currently the main comparison is Table 2 with YOLOv5n, YOLOv8n, YOLOv10n, YOLOv11n, YOLO12n, and YOLO-Net, using only P/R/mAP@0.5.**

Answer: Answer: Thank you for this constructive comment. We agree that the original experimental evaluation could be further strengthened. In the revised manuscript, we have clarified the rationale for selecting the compared YOLO-series lightweight models and have expanded the discussion of the existing results. Since the main objective of this study is to develop a lightweight detector based on YOLO11n for real-time tennis object detection, we focused the primary comparison on models with comparable lightweight deployment characteristics. We also acknowledge that additional metrics such as mAP@0.5:0.95, GFLOPs, FPS, inference time, and class-wise AP would provide a more comprehensive evaluation. We have therefore discussed this limitation more explicitly and will include these metrics in future extended evaluations.

*(Modifications in the manuscript: Discussion sections, pages 25, lines 479-482)*

---------------------------------------------------------------------------------------------------------------------------------

**(3) The dataset is an important asset of the work but is described only briefly.**

Answer: Thank you for this valuable comment. We agree that the dataset is an important component of this work and that the original manuscript did not provide sufficient details regarding its scale, category distribution, and annotation statistics. To address this issue, we have substantially revised the Datasets subsection by adding more detailed information on the dataset construction process, image resolution, scene diversity, target categories, and annotation scale.Specifically, the revised manuscript now states that the dataset contains 6,648 images with a resolution of 1280 × 1280 pixels and includes three object categories: player, tennis racquet, and ball. A total of 21,144 object instances were annotated. The player category appears in 6,434 images with 11,414 annotated instances; the tennis racquet category appears in 4,708 images with 5,754 annotated instances; and the ball category appears in 3,342 images with 3,976 annotated instances. We have also clarified that the dataset covers diverse tennis match scenarios, including different court types, lighting conditions, camera viewpoints, player poses, and background complexities, and that all annotations were manually checked through multiple rounds of quality control.These additions provide a clearer and more comprehensive description of the dataset and better demonstrate its value for evaluating small-object detection in tennis match scenarios.

*(Modifications in the manuscript: Datasets subsection, pages 13–14, lines 259–281.)*

---------------------------------------------------------------------------------------------------------------------------------

**(4) Some essential hyperparameters and design choices are missing or only briefly mentioned.**

Answer: Thank you for this constructive comment. We agree that the original manuscript did not sufficiently clarify how the remaining hyperparameters and implementation settings were handled. To improve reproducibility and fairness, we have revised the Experimental Platform and Parameter Settings subsection by adding a statement that the original YOLO11n default settings were retained for all parameters not explicitly listed in Table 1.

Specifically, the revised manuscript now clarifies that all remaining training hyperparameters, data augmentation settings, loss-related weights, confidence threshold, NMS configuration, and other training and inference settings followed the default YOLO11n configuration and were kept unchanged for all compared models. No additional manual tuning was applied to different comparison models. This ensures that the performance differences mainly reflect the proposed structural improvements rather than differences in hyperparameter adjustment.

*(Modifications in the manuscript: Experimental Platform and Parameter Settings, pages 17, lines 344-349)*

---------------------------------------------------------------------------------------------------------------------------------

**(5) There are several confusing or inconsistent names:**

**The paper’s main method is called YOLO-Net, but:**

**Table 1 uses “YOLO-Net-N/S/M/L/X” although only a single variant is actually discussed.**

**In the Focaler-IoU subsection, “the proposed Soccer-YOLO model” is mentioned (likely a leftover from another paper).**

**In Figure 7 captions and text, the method appears as “Tennis-YOLO” rather than YOLO-Net.**

**Table 2 lists “YOLO12n”, which is not explained (YOLO12 doesn’t exist at the time of writing and is not discussed elsewhere).**

Answer: Thank you for carefully pointing out these naming inconsistencies. We apologize for the confusion caused by these errors. We have thoroughly checked the manuscript and revised the inconsistent names throughout the text, tables, and figure captions.

Specifically, the header “YOLO-Net-N/S/M/L/X” in Table 1 has been replaced with “Value,” since only one YOLO-Net variant is discussed in this study. The incorrect phrase “the proposed Soccer-YOLO model” in the Focaler-IoU subsection has been corrected to “the proposed YOLO-Net model.” In addition, the incorrect label “Tennis-YOLO” in the legend/annotation of Fig. 7 has been corrected to “YOLO-Net,” and the revised figure has been replaced in the manuscript to ensure consistency with the main method name.

Regarding YOLO12n in Table 2, we agree that it was not sufficiently explained in the original manuscript. YOLO12n was included as a recent lightweight YOLO-series baseline in our comparative experiments. YOLOv12 was released in early 2025, whereas this manuscript was prepared in September 2025; therefore, YOLO12n was already available at the time of manuscript preparation and experimental comparison.

*(Modifications in the manuscript: Focaler-IoU subsection, Table 1, Comparative Experiments subsection, Table 2, Figure 7 caption and related tex; pages 14, 17, 18, 19, and 27; lines 297-298, 349, 364-384.)*

---------------------------------------------------------------------------------------------------------------------------------

**(6) The C3k_MSEIS module is central to the claimed edge enhancement, but readers currently rely mostly on equations and a high-level description.**

Answer: Thank you for this constructive comment. We agree that the C3k_MSEIS module is a central component of the proposed YOLO-Net and that the original manuscript relied too heavily on equations and high-level descriptions. To improve clarity and readability, we have revised the C3k_MSEIS subsection by adding a more intuitive explanation of the module motivation, internal data flow, and functional roles of each component.These revisions provide readers with a clearer understanding of how C3k_MSEIS contributes to edge enhancement and small-object detection beyond the mathematical formulations.

*(Modifications in the manuscript: C3k_MSEIS Module subsection, pages 10–11, lines 211–221.)*

---------------------------------------------------------------------------------------------------------------------------------

**(7) Minor comments on language, notation, and formatting.**

Answer: Thank you for this helpful comment. We have carefully revised the manuscript to improve language clarity, notation consistency, and formatting quality. In particular, we corrected typographical and word-breaking errors, standardized the use of technical terms and abbreviations, checked the consistency of model names and module names, and revised figure captions, table titles, and notation where necessary. We have also reviewed the manuscript according to the PLOS ONE formatting requirements.These revisions improve the readability, professionalism, and overall presentation quality of the manuscript.

*(Modifications in the manuscript: throughout the manuscript.)*

**Reviewer 2:**

**The reviewer considered the results interesting and suggested that recent developments in artificial intelligence should be mentioned.**

**Authors propose YOLO-Net, a lightweight detection framework tailored for tennis event analysis. Authors introduces three innovations: a C3k-MSEIS module that enhances multi-scale edge information and dual-domain feature selection to refine fine-grained boundaries; an ECA channel attention mechanism inserted after C2PSA to strengthen inter-channel dependency modeling and improve feature discriminability; and a Focaler-IoU loss function that emphasizes hard and small samples while reducing localization errors. The results are interesting. However, recent development on AI should be mentioned as IEEE Transactions on Intelligent Vehicles 10, 2642-2652, 2025; Engineering Analysis with Boundary Elements 180, 106457, 2025; Expert Systems With Applications 291, 128279, 2025; Physica D 481, 134689, 2025; Physica D 472, 134362, 2025; Nonlinear Dynamics 112, 1291, 2024; Physics of Fluids, 36, 104127, 2024; Physica D 468, 134284, 2024.**

Answer: Thank you for your positive evaluation of our work and for recommending recent studies related to artificial intelligence. We have carefully reviewed and evaluated the suggested publications. We agree that recent developments in AI should be better reflected in the manuscript, particularly in relation to complex dynamic-scene modeling and intelligent perception.

Accordingly, we have revised the Introduction by adding a discussion of recent AI progress beyond conventional computer vision tasks. In particular, we cited the study published in IEEE Transactions on Intelligent Vehicles, which reports graph representation learning for vehicle trajectory prediction in intelligent transportation systems, as it is relevant to AI-based dynamic-scene modeling and intelligent perception. This addition helps broaden the background discussion on recent AI developments before introducing the tennis-specific object detection problem.

For the remaining recommended publications, after careful evaluation, we found that several of them focus mainly on topics such as financial option pricing, physics-informed neural networks for partial differential equation inverse problems, nonlinear dynamics, optical fiber systems, and fluid/traffic state estimation. Although these studies are valuable contributions to AI-related research, they are less directly related to the specific scope of this manuscript, namely lightweight YOLO-based small-object detection in tennis match scenarios. Therefore, we did not include all of them as direct citations, in accordance with the editor’s clarification that there is no requirement to cite recommended works unless they are directly relevant.

*(Modifications in the manuscript: Introduction section and References, page 3, 27, lines 50–52, 533-535.)*

**Reviewer 3:**

The reviewer recommended major revision and raised important concerns regarding manuscript quality, experimental completeness, novelty, related work, ablation design, and qualitative results.

**(1) There are noticeable word-breaking and formatting problems, such as “fi-ne” and “re-al-time,” which should be corrected for clarity and professionalism.**

Answer: Thank you for this helpful comment. We have carefully checked the entire manuscript and corrected the word-breaking and formatting problems caused by improper hyphenation, line breaks, and text insertion. For example, terms such as “con-taining,” “outperform-ing,” “re-al-time,” and “in-telligent” have been corrected to “containing,” “outperforming,” “real-time,” and “intelligent,” respectively. We also fixed punctuation and notation issues to improve readability and professional presentation.

*(Modifications in the manuscript: Abstract, pages 3, lines 27-33)*

---------------------------------------------------------------------------------------------------------------------------------

**(2) Several abbreviations are introduced without their full names at first occurrence, such as YOLO, DETR, Faster R-CNN, and SSD.**

Answer: Thank you for this helpful comment. We have carefully checked the manuscript and revised the first occurrence of abbreviations to include their full names. For example, YOLO has been revised to “You Only Look Once (YOLO),” DETR to “Detection Transformer (DETR),” Faster R-CNN to “Faster Region-based Convolutional Neural Network (Faster R-CNN),” and SSD to “Single Shot MultiBox Detector (SSD).” These revisions improve the clarity and readability of the manuscript, especially for readers who may not be familiar with all object detection abbreviations.

*(Modifications in the manuscript: Introduction, pages 3, lines 53-55.)*

---------------------------------------------------------------------------------------------------------------------------------

**(3) The comparison experiments in this paper are relatively limited. The proposed method is mainly compared with baseline models such as the original YOLO variants (e.g., YOLOv5, YOLOv8, YOLO11n), which are not specifically optimized for small-object detection. However, a large number of recent studies have proposed improved YOLO-based methods tailored for small-object scenarios. It is recommended that the authors include comparisons with these state-of-the-art improved YOLO models (especially recent variants, e.g., YOLO13, YOLO2026, or other small-object-oriented improvements) to better demonstrate the effectiveness and competitiveness of the proposed method.**

Answer: Thank you for this constructive suggestion. We agree that broader comparisons with recent YOLO variants and small-object-oriented detectors can further strengthen the experimental evaluation. To address this comment, we have expanded the comparative experiments by adding YOLO26n as a recent lightweight YOLO-series baseline. The revised Table 2 now compares YOLO-Net with YOLOv5n, YOLOv8n, YOLOv10n, YOLO11n, YOLO12n, and YOLO26n under the same tennis dataset and training protocol.

The new comparison shows that although YOLO26n has the smallest parameter count among the compared models, YOLO-Net achieves the highest Precision and mAP@0.5. Specifically, YOLO-Net obtains 84.5% Precision and 78.2% mAP@0.5 with 2.58M parameters, while YOLO26n obtains 83.6% Precision and 77.9% mAP@0.5 with 2.40M parameters. These results further demonstrate that YOLO-Net provides a favorable balance between detection accuracy and model complexity for tennis small-object detection.

Regarding other improved YOLO-based small-object detectors, many of them are designed for specific domains such as remote sensing, UAV imagery, traffic monitoring, medical detection, agricultural disease detection, or industrial defects. Their network structures, training strategies, and target characteristics are often optimized for those specific datasets and may not be directly comparable to tennis match scenarios involving players, racquets, and fast-moving tennis balls. Therefore, in the current revision, we focused on stable and representative lightweight YOLO-series models under a unified training and evaluation protocol. We have also added a related-work discussion on recent YOLO-based module-combination methods for small-object detection and clarified this limitation in the manuscript.

*(Modifications in the manuscript: Comparative Experiments, pages 18–19, lines 364–384.)*

---------------------------------------------------------------------------------------------------------------------------------

**(4) The novelty of the proposed method requires further clarification. The ECA attention mechanism and Focaler-CIoU loss are existing techniques, and this work mainly integrates them into the YOLO11 framework. It is therefore unclear whether similar integrations have already been explored in prior studies, especially in small-object detection scenarios.**

Answer: Thank you for this insightful comment. We agree that the novelty of the proposed method should be clarified more carefully, especially because ECA attention and Focaler-IoU are existing techniques. We did not intend to claim that ECA or Focaler-IoU themselves are newly proposed in this work. To avoid this misunderstanding, we have revised the Discussion section to explicitly state that ECA attention and Focaler-IoU have been explored in previous studies and are adopted as complementary components in YOLO-Net.

Specifically, we clarified that the main structural contribution of this work is the C3k_MSEIS module, which introduces edge-aware multi-scale feature enhancement into the YOLO11n backbone. ECA is used to refine channel-level discriminability, while Focaler-IoU is adopted to improve bounding-box regression for small and hard samples. Their integration is task-oriented rather than purely additive and is designed specifically for tennis match scenarios involving small fast-moving balls, racquet occlusion, motion blur, and complex backgrounds.

*(Modifications in the manuscript: Discussion section, page 24-25, lines 363–375.)*

---------------------------------------------------------------------------------------------------------------------------------

**(5) Please provide a more comprehensive review of related work on such module combinations and clearly position the contribution relative to existing approaches.**

Answer: Thank you for this valuable suggestion. We agree that the original Related Work section did not sufficiently review existing YOLO-based module-combination strategies. To address this issue, we have added a new subsection entitled “YOLO-Based Module Combinations for Small-Object Detection” in the Related Work section.

In this new subsection, we reviewed recent studies that improve YOLO-based detectors by combining attention mechanisms, multi-scale feature enhancement, lightweight convolutional modules, additional small-object detection layers, dynamic upsampling, and optimized bounding-box regression losses. We also summarized the main purposes of these modules, including strengthening effective target features, suppressing background interference, improving small-object representation, reducing model complexity, and improving localization accuracy for hard samples. Furthermore, we discussed the limitations of existing module-combination methods. Many of them are designed for specific domains such as remote sensing, UAV imagery, traffic scenes, medical detection, or industrial defects, and their target characteristics differ from tennis match scenarios. Some methods also rely on directly stacking attention modules, extra detection heads, or feature-fusion blocks, which may increase computational complexity and weaken the real-time advantage of lightweight YOLO detectors. Based on this review, we clarified the positioning of YOLO-Net. Compared with these general module-combination strategies, YOLO-Net focuses on tennis-specific small-object detection and emphasizes edge-aware multi-scale feature enhancement under lightweight deployment constraints. These revisions better distinguish the proposed method from existing YOLO-based improvement strategies and clarify its task-oriented contribution.

*(Modifications in the manuscript: Related Work section, pages 7-8, lines 144–169.)*

---------------------------------------------------------------------------------------------------------------------------------

**(6) The ablation study is relatively limited and does not convincingly demonstrate the individual contributions of each proposed component. The current design follows a purely sequential accumulation, which makes it difficult to disentangle the effect of each module. In particular, key variants such as baseline+ECA, baseline+Focaler-CIoU, and other pairwise combinations are missing.**

Answer: Thank you for this constructive comment. We understand the reviewer’s concern regarding the ablation design. In this study, the ablation experiments were designed as a progressive accumulation process, which is commonly used to evaluate the performance changes introduced by each module along the actual construction path of the proposed model. Specifically, the baseline YOLO11n was first used as Model A, C3k_MSEIS was then added as Model B, ECA was further added as Model C, and Focaler-IoU was finally introduced as Model D. This progressive design allows us to observe the incremental contribution of each component to the final YOLO-Net framework. In addition, we have provided separate comparative experiments for individual module choices, including comparisons among different attention mechanisms and different IoU loss functions, which further support the effectiveness of ECA and Focaler-IoU, respectively.

---------------------------------------------------------------------------------------------------------------------------------

**(7) The qualitative results do not convincingly demonstrate the challenging scenarios claimed in the paper. Fig.5 mainly shows standard domain variations (e.g., court color and background), rather than truly difficult cases such as small targets, motion blur, or occlusion. Similarly, Fig.7 presents mostly clean and simple scenes, where both methods perform well, lacking hard examples or failure cases to highlight the advantages of the proposed method. It is recommended to include more representative challenging scenarios to better validate the effectiveness of the approach.**

Answer: Thank you for this constructive comment. We understand the reviewer’s concern that the challenging aspects shown in the qualitative figures were not sufficiently highlighted in the original manuscript. Figure 5 is intended to present representative dataset samples rather than detection outputs. We have revised the corresponding description to clarify that the dataset contains long-distance views, small tennis-ball targets, different court colors, varying camera perspectives, and complex match backgrounds, which together reflect the diversity and difficulty of the constructed dataset.For Figure 7, we have revised the figure annotation and textual explanation to more explicitly describe the challenging detection cases. In particular, the figure includes examples of false detection, small-ball detection under long-distance views, and confidence-score improvement achieved by YOLO-Net compared with YOLO11. The upper row illustrates detection errors produced by the baseline model, while the lower row shows that YOLO-Net improves the confidence and stability of small-ball detection in the same scenes. We have also corrected the label in Fig. 7 from “Tennis-YOLO” to “YOLO-Net” to maintain consistency with the method name.These revisions make the qualitative analysis clearer and better demonstrate the performance of YOLO-Net under challenging tennis match scenarios.

*(Modifications in the manuscript: Dataset description, Figure 7, and qualitative analysis text, pages 16, 19, lines 327-331, 374-384.)*
