# Supplementary material for: YOLO-Net: A lightweight edge-enhanced detection model for small-object recognition in tennis match scenarios
Source: PLoS One. 2026 Jul 14;21(7):e0335558. doi: 10.1371/journal.pone.0335558 (PMC13367711; doi:10.1371/journal.pone.0335558)
Supplement: Peer review history

# Peer review: Decision Letter 0

**DOI:** 10.1371/journal.pone.0335558.r001

**Authors:** Brian Weaver

28 Apr 2026

PONE-D-25-55470

YOLO-Net: A lightweight edge-enhanced detection model for small-object recognition in tennis match scenarios

PLOS One

Dear Dr. Jia,

Thank you for submitting your manuscript to PLOS ONE. After careful consideration, we feel that it has merit but does not fully meet PLOS ONE’s publication criteria as it currently stands. Therefore, we invite you to submit a revised version of the manuscript that addresses the points raised during the review process.

The manuscript has been evaluated by three reviewers, and their comments are available below. The reviewers have raised a number of concerns with the methodology in your manuscript that need attention. Could you please revise the manuscript to carefully address the concerns raised?

Please note that comments from reviewer 2 include recommendations to cite specific previously published works, please review and evaluate these publications to determine whether they are relevant. There is no requirement to cite these works.

Please submit your revised manuscript by Jun 08 2026 11:59PM. If you will need more time than this to complete your revisions, please reply to this message or contact the journal office at plosone@plos.org. When you're ready to submit your revision, log on to https://www.editorialmanager.com/pone/ and select the 'Submissions Needing Revision' folder to locate your manuscript file.

Please include the following items when submitting your revised manuscript:

- A letter that responds to each point raised by the academic editor and reviewer(s). You should upload this letter as a separate file labeled 'Response to Reviewers'.
- A marked-up copy of your manuscript that highlights changes made to the original version. You should upload this as a separate file labeled 'Revised Manuscript with Track Changes'.
- An unmarked version of your revised paper without tracked changes. You should upload this as a separate file labeled 'Manuscript'.

If you would like to make changes to your financial disclosure, please include your updated statement in your cover letter. Guidelines for resubmitting your figure files are available below the reviewer comments at the end of this letter.

If applicable, we recommend that you deposit your laboratory protocols in protocols.io to enhance the reproducibility of your results. Protocols.io assigns your protocol its own identifier (DOI) so that it can be cited independently in the future. For instructions see: https://journals.plos.org/plosone/s/submission-guidelines#loc-laboratory-protocols. Additionally, PLOS ONE offers an option for publishing peer-reviewed Lab Protocol articles, which describe protocols hosted on protocols.io. Read more information on sharing protocols at https://plos.org/protocols?utm_medium=editorial-email&utm_source=authorletters&utm_campaign=protocols.

As the corresponding author, your ORCID iD is verified in the submission system and will appear in the published article. PLOS supports the use of ORCID, and we encourage all coauthors to register for an ORCID iD and use it as well. Please encourage your coauthors to verify their ORCID iD within the submission system before final acceptance, as unverified ORCID iDs will not appear in the published article. *Only*  the individual author can complete the verification step; PLOS staff *cannot*  verify ORCID iDs on behalf of authors.

We look forward to receiving your revised manuscript.

Kind regards,

Brian Patrick Weaver, Ph.D.

Staff Editor

PLOS One

Journal Requirements:

When submitting your revision, we need you to address these additional requirements.

1. Please ensure that your manuscript meets PLOS ONE's style requirements, including those for file naming. The PLOS ONE style templates can be found at

https://journals.plos.org/plosone/s/file?id=wjVg/PLOSOne_formatting_sample_main_body.pdf and

https://journals.plos.org/plosone/s/file?id=ba62/PLOSOne_formatting_sample_title_authors_affiliations.pdf.

2. In your Methods section, please include additional information about your dataset and ensure that you have included a statement specifying whether the collection and analysis method complied with the terms and conditions for the source of the data.

3. Please note that PLOS One has specific guidelines on code sharing for submissions in which author-generated code underpins the findings in the manuscript. In these cases, we expect all author-generated code to be made available without restrictions upon publication of the work. Please review our guidelines at https://journals.plos.org/plosone/s/materials-and-software-sharing#loc-sharing-code and ensure that your code is shared in a way that follows best practice and facilitates reproducibility and reuse.

4. Thank you for stating in your Funding Statement:

“This work was supported by the MOE (Ministry of Education in China) Liberal Arts and Social Sciences Foundation under Grant 21YJCZH191 “

Please provide an amended statement that declares *all* the funding or sources of support (whether external or internal to your organization) received during this study, as detailed online in our guide for authors at http://journals.plos.org/plosone/s/submit-now.  Please also include the statement “There was no additional external funding received for this study.” in your updated Funding Statement.

Please include your amended Funding Statement within your cover letter. We will change the online submission form on your behalf.

5. Thank you for stating the following financial disclosure:

“This work was supported by the MOE (Ministry of Education in China) Liberal Arts and Social Sciences Foundation under Grant 21YJCZH191 “

Please state what role the funders took in the study.  If the funders had no role, please state: "The funders had no role in study design, data collection and analysis, decision to publish, or preparation of the manuscript."

If this statement is not correct you must amend it as needed.

Please include this amended Role of Funder statement in your cover letter; we will change the online submission form on your behalf.

6. We note that you have indicated that there are restrictions to data sharing for this study. PLOS only allows data to be available upon request if there are legal or ethical restrictions on sharing data publicly. For more information on unacceptable data access restrictions, please see http://journals.plos.org/plosone/s/data-availability#loc-unacceptable-data-access-restrictions.

Before we proceed with your manuscript, please address the following prompts:

a) If there are ethical or legal restrictions on sharing a de-identified data set, please explain them in detail (e.g., data contain potentially identifying or sensitive patient information, data are owned by a third-party organization, etc.) and who has imposed them (e.g., a Research Ethics Committee or Institutional Review Board, etc.). Please also provide contact information for a data access committee, ethics committee, or other institutional body to which data requests may be sent.

b) If there are no restrictions, please upload the minimal anonymized data set necessary to replicate your study findings to a stable, public repository and provide us with the relevant URLs, DOIs, or accession numbers. For a list of recommended repositories, please see

https://journals.plos.org/plosone/s/recommended-repositories. You also have the option of uploading the data as Supporting Information files, but we would recommend depositing data directly to a data repository if possible.

We will update your Data Availability statement on your behalf to reflect the information you provide.

7.In the online submission form you indicate that your data is not available for proprietary reasons and have provided a contact point for accessing this data. Please note that your current contact point is a co-author on this manuscript. According to our Data Policy, the contact point must not be an author on the manuscript and must be an institutional contact, ideally not an individual. Please revise your data statement to a non-author institutional point of contact, such as a data access or ethics committee, and send this to us via return email. Please also include contact information for the third party organization, and please include the full citation of where the data can be found.

8. PLOS requires an ORCID iD for the corresponding author in Editorial Manager on papers submitted after December 6th, 2016. Please ensure that you have an ORCID iD and that it is validated in Editorial Manager. To do this, go to ‘Update my Information’ (in the upper left-hand corner of the main menu), and click on the Fetch/Validate link next to the ORCID field. This will take you to the ORCID site and allow you to create a new iD or authenticate a pre-existing iD in Editorial Manager.

9. Please ensure that you refer to Figure 8 in your text as, if accepted, production will need this reference to link the reader to the figure

10. If the reviewer comments include a recommendation to cite specific previously published works, please review and evaluate these publications to determine whether they are relevant and should be cited. There is no requirement to cite these works unless the editor has indicated otherwise.

[Note: HTML markup is below. Please do not edit.]

Reviewers' comments:

Reviewer's Responses to Questions

**Comments to the Author**

1. Is the manuscript technically sound, and do the data support the conclusions?

The manuscript must describe a technically sound piece of scientific research with data that supports the conclusions. Experiments must have been conducted rigorously, with appropriate controls, replication, and sample sizes. The conclusions must be drawn appropriately based on the data presented.

Reviewer #1: Yes

Reviewer #2: Yes

Reviewer #3: Partly

**********

2. Has the statistical analysis been performed appropriately and rigorously?

Reviewer #1: N/A

Reviewer #2: Yes

Reviewer #3: No

**********

3. Have the authors made all data underlying the findings in their manuscript fully available?

The PLOS Data policy requires authors to make all data underlying the findings described in their manuscript fully available without restriction, with rare exception (please refer to the Data Availability Statement in the manuscript PDF file). The data should be provided as part of the manuscript or its supporting information, or deposited to a public repository. For example, in addition to summary statistics, the data points behind means, medians and variance measures should be available. If there are restrictions on publicly sharing data—e.g. participant privacy or use of data from a third party—those must be specified.

Reviewer #1: Yes

Reviewer #2: No

Reviewer #3: No

**********

4. Is the manuscript presented in an intelligible fashion and written in standard English?

PLOS ONE does not copyedit accepted manuscripts, so the language in submitted articles must be clear, correct, and unambiguous. Any typographical or grammatical errors should be corrected at revision, so please note any specific errors here.

Reviewer #1: Yes

Reviewer #2: Yes

Reviewer #3: No

**********

5. Review Comments to the Author

Please use the space provided to explain your answers to the questions above. You may also include additional comments for the author, including concerns about dual publication, research ethics, or publication ethics. (Please upload your review as an attachment if it exceeds 20,000 characters)

Reviewer #1: Addresses a real and relevant application: small-object detection (tennis ball, racquet) in broadcast-style tennis videos.

Proposes a reasonably coherent set of improvements to YOLO11n.

There are some major issues:

1. Clarify the exact novelty and contribution. Right now, the work looks like a well-engineered combination rather than a fundamentally new detection architecture.

2. Experimental evaluation needs to be strengthened. Currently the main comparison is Table 2 (YOLOv5n, v8n, v10n, v11n, “YOLO12n”, YOLO-Net) with P/R/mAP@0.5 only.

3. The dataset is an important asset of the work but is described only briefly.

4. Some essential hyperparameters and design choices are missing or only briefly mentioned.

5. There are several confusing or inconsistent names:

The paper’s main method is called YOLO-Net, but:

Table 1 uses “YOLO-Net-N/S/M/L/X” although only a single variant is actually discussed.

In the Focaler-IoU subsection, “the proposed Soccer-YOLO model” is mentioned (likely a leftover from another paper).

In Figure 7 captions and text, the method appears as “Tennis-YOLO” rather than YOLO-Net.

Table 2 lists “YOLO12n”, which is not explained (YOLO12 doesn’t exist at the time of writing and is not discussed elsewhere).

6. The C3k_MSEIS module is central to the claimed edge enhancement, but readers currently rely mostly on equations and a high-level description.

7. Minor comments (language, notation, formatting).

Reviewer #2: Authors propose YOLO-Net, a lightweight detection framework tailored for tennis event analysis. Authors introduces three innovations: a C3k-MSEIS module that enhances multi-scale edge information and dual-domain feature selection to refine fi-ne-grained boundaries; an ECA channel attention mechanism inserted after C2PSA to strengthen inter-channel dependency modeling and improve feature discriminability; and a Focaler-IoU loss function that emphasizes hard and small samples while reducing lo-calization errors. The results are interesting. However, recent development on AI should be mentioned as IEEE Transactions on Intelligent Vehicles 10, 2642-2652, 2025;Engineering Analysis with Boundary Elements 180, 106457, 2025;Expert Systems With Applications 291, 128279, 2025;Physica D 481, 134689, 2025; Physica D 472, 134362, 2025;Nonlinear Dynamics 112, 1291, 2024;Physics of Fluids, 36, 104127, 2024;Physica D 468, 134284, 2024.

Reviewer #3: The paper proposes YOLO-Net, a lightweight detection framework built upon YOLO11n with the integration of C3k-MSEIS, ECA attention, and Focaler-IoU loss. The study is application-oriented, and the results demonstrate a reasonable balance between accuracy and model efficiency. However, several important issues remain in terms of manuscript quality, experimental completeness and novelty. Therefore, major revision is recommended.

1. There are noticeable word-breaking and formatting problems (e.g., “fi-ne”, “re-al-time”), which should be corrected for clarity and professionalism.

2. The paper contains several abbreviations that are introduced without their full names at first occurrence (e.g., YOLO, DETR, Faster R-CNN, SSD).

3. The comparison experiments in this paper are relatively limited. The proposed method is mainly compared with baseline models such as the original YOLO variants (e.g., YOLOv5, YOLOv8, YOLO11n), which are not specifically optimized for small-object detection. However, a large number of recent studies have proposed improved YOLO-based methods tailored for small-object scenarios. It is recommended that the authors include comparisons with these state-of-the-art improved YOLO models (especially recent variants, e.g., YOLO13, YOLO2026, or other small-object-oriented improvements) to better demonstrate the effectiveness and competitiveness of the proposed method.

4. The novelty of the proposed method requires further clarification. The ECA attention mechanism and Focaler-CIoU loss are existing techniques, and this work mainly integrates them into the YOLO11 framework. It is therefore unclear whether similar integrations have already been explored in prior studies, especially in small-object detection scenarios.

5. Please provide a more comprehensive review of related work on such module combinations and clearly position their contribution relative to existing approaches.

6. The ablation study is relatively limited and does not convincingly demonstrate the individual contributions of each proposed component. The current design follows a purely sequential accumulation, which makes it difficult to disentangle the effect of each module. In particular, key variants such as baseline+ECA, baseline+Focaler-CIoU, and other pairwise combinations are missing.

7. The qualitative results do not convincingly demonstrate the challenging scenarios claimed in the paper. Fig.5 mainly shows standard domain variations (e.g., court color and background), rather than truly difficult cases such as small targets, motion blur, or occlusion. Similarly, Fig.7 presents mostly clean and simple scenes, where both methods perform well, lacking hard examples or failure cases to highlight the advantages of the proposed method. It is recommended to include more representative challenging scenarios to better validate the effectiveness of the approach.

**********

6. PLOS authors have the option to publish the peer review history of their article (what does this mean?). If published, this will include your full peer review and any attached files.

If you choose “no”, your identity will remain anonymous but your review may still be made public.

**Do you want your identity to be public for this peer review?**  For information about this choice, including consent withdrawal, please see our Privacy Policy.

Reviewer #1: No

Reviewer #2: No

Reviewer #3: No

**********

[NOTE: If reviewer comments were submitted as an attachment file, they will be attached to this email and accessible via the submission site. Please log into your account, locate the manuscript record, and check for the action link "View Attachments". If this link does not appear, there are no attachment files.]

To ensure your figures meet our technical requirements, please review our figure guidelines: https://journals.plos.org/plosone/s/figures

You may also use PLOS’s free figure tool, NAAS, to help you prepare publication quality figures: https://journals.plos.org/plosone/s/figures#loc-tools-for-figure-preparation.

NAAS will assess whether your figures meet our technical requirements by comparing each figure against our figure specifications.

# Author comment: Author response to Decision Letter 1

**DOI:** 10.1371/journal.pone.0335558.r002

2 Jun 2026

Dear Editors and Reviewers,

Thank you for your constructive comments and suggestions. We have carefully revised the manuscript according to the editorial requirements and reviewer comments. A detailed point-by-point response to all comments has been provided in the uploaded Response to Reviewers document. The revised manuscript, revised cover letter, and revised figure files have also been uploaded.

Sincerely,

Liangquan Jia

On behalf of all authors

Attachment Submitted filename: Response to Reviewers.docx

# Peer review: Decision Letter 1

**DOI:** 10.1371/journal.pone.0335558.r003

**Authors:** Brian Weaver, Gen Li

23 Jun 2026

YOLO-Net: A lightweight edge-enhanced detection model for small-object recognition in tennis match scenarios

PONE-D-25-55470R1

Dear Dr. Jia,

We’re pleased to inform you that your manuscript has been judged scientifically suitable for publication and will be formally accepted for publication once it meets all outstanding technical requirements.

Within one week, you’ll receive an e-mail detailing the required amendments. When these have been addressed, you’ll receive a formal acceptance letter and your manuscript will be scheduled for publication.

An invoice will be generated when your article is formally accepted. Please note, if your institution has a publishing partnership with PLOS and your article meets the relevant criteria, all or part of your publication costs will be covered. Please make sure your user information is up-to-date by logging into Editorial Manager at Editorial Manager® and clicking the ‘Update My Information' link at the top of the page. For questions related to billing, please contact billing support.

If your institution or institutions have a press office, please notify them about your upcoming paper to help maximize its impact. If they’ll be preparing press materials, please inform our press team as soon as possible -- no later than 48 hours after receiving the formal acceptance. Your manuscript will remain under strict press embargo until 2 pm Eastern Time on the date of publication. For more information, please contact onepress@plos.org.

Kind regards,

Gen Li

Academic Editor

PLOS One

Additional Editor Comments (optional):

Reviewers' comments:

Reviewer's Responses to Questions

**Comments to the Author**

1. If the authors have adequately addressed your comments raised in a previous round of review and you feel that this manuscript is now acceptable for publication, you may indicate that here to bypass the “Comments to the Author” section, enter your conflict of interest statement in the “Confidential to Editor” section, and submit your "Accept" recommendation.

Reviewer #1: All comments have been addressed

Reviewer #3: All comments have been addressed

**********

2. Is the manuscript technically sound, and do the data support the conclusions?

The manuscript must describe a technically sound piece of scientific research with data that supports the conclusions. Experiments must have been conducted rigorously, with appropriate controls, replication, and sample sizes. The conclusions must be drawn appropriately based on the data presented.

Reviewer #1: Yes

Reviewer #3: Partly

**********

3. Has the statistical analysis been performed appropriately and rigorously?

Reviewer #1: Yes

Reviewer #3: Yes

**********

4. Have the authors made all data underlying the findings in their manuscript fully available?

The PLOS Data policy requires authors to make all data underlying the findings described in their manuscript fully available without restriction, with rare exception (please refer to the Data Availability Statement in the manuscript PDF file). The data should be provided as part of the manuscript or its supporting information, or deposited to a public repository. For example, in addition to summary statistics, the data points behind means, medians and variance measures should be available. If there are restrictions on publicly sharing data—e.g. participant privacy or use of data from a third party—those must be specified.

Reviewer #1: Yes

Reviewer #3: Yes

**********

5. Is the manuscript presented in an intelligible fashion and written in standard English?

PLOS ONE does not copyedit accepted manuscripts, so the language in submitted articles must be clear, correct, and unambiguous. Any typographical or grammatical errors should be corrected at revision, so please note any specific errors here.

Reviewer #1: Yes

Reviewer #3: Yes

**********

6. Review Comments to the Author

Please use the space provided to explain your answers to the questions above. You may also include additional comments for the author, including concerns about dual publication, research ethics, or publication ethics. (Please upload your review as an attachment if it exceeds 20,000 characters)

Reviewer #1: The manuscript presents a well-executed study on lightweight object detection for tennis match scenarios. The proposed YOLO-Net effectively integrates multi-scale edge enhancement, channel attention, and improved localization loss to address the challenges associated with small and fast-moving objects. The methodology is clearly described, and the experimental results, including ablation studies and comparative analyses, provide convincing evidence of the effectiveness of the proposed approach. The manuscript is well organized, the figures and tables are informative, and the discussion appropriately acknowledges the limitations and future research directions. Overall, the work makes a meaningful contribution to sports analytics and real-time object detection research. I commend the authors for their thorough revisions and believe the manuscript is suitable for publication in its current form.

Reviewer #3: The authors have addressed all my previous comments thoroughly, and the manuscript has been significantly improved in quality. I recommend acceptance.

**********

7. PLOS authors have the option to publish the peer review history of their article (what does this mean?). If published, this will include your full peer review and any attached files.

If you choose “no”, your identity will remain anonymous but your review may still be made public.

**Do you want your identity to be public for this peer review?**  For information about this choice, including consent withdrawal, please see our Privacy Policy.

Reviewer #1: **Yes:** Dr. Vipal Kumar Sharma

Reviewer #3: No

**********

# Editor report: Acceptance letter

**DOI:** 10.1371/journal.pone.0335558.r004

**Authors:** Brian Weaver, Gen Li

PONE-D-25-55470R1

PLOS One

Dear Dr. Jia,

I'm pleased to inform you that your manuscript has been deemed suitable for publication in PLOS One. Congratulations! Your manuscript is now being handed over to our production team.

At this stage, our production department will prepare your paper for publication. This includes ensuring the following:

* All references, tables, and figures are properly cited

* All relevant supporting information is included in the manuscript submission,

* There are no issues that prevent the paper from being properly typeset

You will receive further instructions from the production team, including instructions on how to review your proof when it is ready. Please keep in mind that we are working through a large volume of accepted articles, so please give us a few days to review your paper and let you know the next and final steps.

Lastly, if your institution or institutions have a press office, please let them know about your upcoming paper now to help maximize its impact. If they'll be preparing press materials, please inform our press team within the next 48 hours. Your manuscript will remain under strict press embargo until 2 pm Eastern Time on the date of publication. For more information, please contact onepress@plos.org.

You will receive an invoice from PLOS for your publication fee after your manuscript has reached the completed accept phase. If you receive an email requesting payment before acceptance or for any other service, this may be a phishing scheme. Learn how to identify phishing emails and protect your accounts at https://explore.plos.org/phishing.

If we can help with anything else, please email us at customercare@plos.org.

Thank you for submitting your work to PLOS ONE and supporting open access.

Kind regards,

PLOS ONE Editorial Office Staff

on behalf of

Dr. Gen Li

Academic Editor

PLOS One
